# Is the association between high strain work and depressive symptoms modified by private life social support: a cohort study of 1,074 Danish employees?

**DOI:** 10.1186/1471-2458-14-698

**Published:** 2014-07-08

**Authors:** Ida EH Madsen, Anette FB Jorgensen, Marianne Borritz, Martin L Nielsen, Reiner Rugulies

**Affiliations:** 1 National Research Centre for the Working Environment, Lerso Parkalle 105, DK-2100 Copenhagen, Denmark; 2 Occupational Medicine Department, Koge Hospital, Lykkebaekvej 1, DK-4600, Koge, Denmark; 3 Department of Occupational and Environmental Medicine, Bispebjerg University Hospital, Bispebjerg Bakke 23, DK-2400 Copenhagen, Denmark; 4 Department of Public Health, University of Copenhagen, Oster Farimagsgade 5, DK-1014 Copenhagen, Denmark; 5 Department of Psychology, University of Copenhagen, Oster Farimagsgade 2A, DK-1353 Copenhagen, Denmark

**Keywords:** Depression, Work stress, Psychosocial factors, Interaction, Occupational health

## Abstract

**Background:**

Previous studies have shown that psychosocial working conditions characterized by high psychological demands and low decision latitude (i.e., high strain work) are associated with increased risk of depressive symptoms. Little is known, however, concerning how this association may be modified by factors outside the working environment. This article examines the modifying role of private life social support in the relation between high strain work and the development of severe depressive symptoms.

**Methods:**

Data were questionnaire-based, collected from a cross-occupational sample of 1,074 Danish employees. At baseline, all participants were free of severe depressive symptoms, measured by the Mental Health Inventory. High strain work was defined by the combination of high psychological demands at work and low control, measured with multi-dimensional scales. Private life social support was operationalized as the number of life domains with confidants and dichotomized as low (0–1 domains) or high (2 or more domains). Using logistic regression we examined the risk of onset of severe depressive symptoms, adjusting for sex, age, occupational position, and prior depressive symptoms.

**Results:**

Separately, neither high strain work nor low private life social support statistically significantly predicted depressive symptoms. However, participants with joint exposure to high strain work and low private life social support had an Odds ratio (OR) for severe depressive symptoms of 3.41 (95% CI: 1.36-8.58), compared to participants with no work strain and high private life social support. There was no increased risk for participants with high strain work and high private life social support (OR = 1.32, 95% CI: 0.65-2.68). The interaction term for departure from additivity was, however, not statistically significant (p = 0.18).

**Conclusions:**

Our findings suggest that high strain work may increase risk of depressive symptoms in individuals with low private life social support, although the effect-modification was statistically non-significant. Larger studies are needed to further establish the role of private life social support in the relation between high strain work and depression.

## Background

Depression is associated with substantial costs, both for the individuals affected and society at large [1,2]. The etiology of depression is thought to involve a complex interplay of biological, psychological and social factors [3-5]. Psychosocial factors within and beyond the work environment have previously been associated with depression in working populations [6-9]. Little is known, however, concerning their interplay, and whether non-work-related factors may modify the association between the working environment and the development of depression [9,10]. The current knowledge gap regarding the interplay between work-related and non-work-related factors may result in both over- and underestimations of the impact of the working environment, by averaging across dissimilar groups.

The demands-control model states that the risk of stress-related disorders is increased amongst employees with a psychosocial work environment characterized by high psychological demands and low decision latitude (i.e., high strain work) [11]. Numerous studies have examined the associations between high strain work and depression and at least three systematic reviews have found that there is good evidence of a prospective relation [6-8]. Following Hobfoll’s conservation of resources theory, it is plausible that the consequences of the resource expenditure elicited by high strain work depend on the availability of resources [12]. Social support is an important resource [12], and may buffer the effects of encountered strains emotionally, by sustaining the individual’s self-worth, sense of mattering, and feelings of belonging and acceptance [13,14]. Social support may also take the form of instrumental aid, information and advice, and encouragement to cope with the situation; all factors which might help the individual cope effectively with any strain experienced [13,14]. Regarding high strain work, evidence is building that this exposure is particularly detrimental in the context of low social support at work (so called iso strain) [15]. Evidence is scarce, however, regarding whether the health consequences of high strain work might depend on the availability of resources outside the workplace [10].

In this study we examined if the prospective association between high strain work and onset of severe depressive symptoms is modified by levels of private life social support. More specifically, we assess whether the risk of severe depressive symptoms associated with the joint exposure to both high strain work and low private life social support is greater than the sum of its separate parts, indicating synergistic effects [16].

## Methods

This study used a cohort-design, analysing data from two existing Danish work environment studies conducted during 1996–2005, namely the Project on Burnout, Motivation and Job Satisfaction (Danish Acronym: PUMA) and The Intervention Project on Absence and Well-being (IPAW). The studies contained similar data on select exposures and were combined to increase the sample size. Details of the studies are published elsewhere [17,18]. In brief, PUMA was a an open cohort intervention study designed to examine burn-out amongst human service workers with three waves of data-collection in 1999–2000, 2002–2003, and 2005. Participants in PUMA were recruited from 7 human service organizations in Danish social work, health care work, elder care work, and care work dealing with handicapped persons, prison officers and prison professionals (in a psychiatric prison). Of the 2,391 individuals initially invited, there were 1,941 respondents to the initial data-collection in PUMA (response rate = 80%). The IPAW study was also a three-wave open cohort intervention study conducted to improve well-being and thereby reduce sickness absence rates in a pharmaceutical company, the municipal technical services and the municipal nursing homes in Copenhagen, Denmark. The three waves of IPAW data used in the present study were collected in 1996–1997, 1997–1998, and 2001–2002. Of the 2,721 individuals initially invited, there were 2,068 respondents to the initial data-collection in IPAW (response rate = 76%).

This study was conducted according to the Helsinki declaration. All participants were informed in the questionnaires about the purpose of the study, and consented by filling in and returning the questionnaires. This study was approved by and registered with the Danish data protection agency [19]. According to Danish law, studies involving only questionnaire or register data are not required to obtain approval by the scientific ethical committee’s [20]. For the original PUMA study, ethical approval was obtained from the regional ethical committee’s [17], whereas the IPAW study team was informed by the ethical committee that the case would not be considered, as data were purely questionnaire and register based and no approval was required.

### Selection of participants

Both PUMA and IPAW encompassed three waves of data-collection. We use wave 2 data as baseline to enable statistical control for prior depressive symptoms (measured at wave 1), as a proxy for negative affectivity [21]. We used wave 1 data for this control to ensure the correct temporal sequence and avoid adjusting for a potential mediator such as baseline depressive symptoms. For the present analyses we selected the 1,258 individuals who participated in all three waves of the two studies, but excluded individuals with wave 2 severe depressive symptoms (n = 111) to examine prospective associations between exposures measured at wave 2 and onset of severe depressive symptoms measured at wave 3. We further excluded 73 individuals with missing data on exposure, outcome or potential confounders, yielding a final sample size of 1,074.

### Measurements

Data were collected by questionnaire. High strain work was defined as the combination of high psychological demands and low decision latitude, measured with harmonized multidimensional scales based on the items presented in Table 1. The measurement of psychological demands comprised the dimensions of quantitative and conflicting demands, and the measurement of decision latitude included both skill discretion and decision authority in accordance with the demands control model [11]. Each item was scored from 1 to 5 in PUMA and 1 to 4 in IPAW, and the scale was calculated based on an equally weighted mean if half or more items were completed. The scales were then standardized to a mean of 0 and a standard deviation of 1, and dichotomized into high and low by median split. The applied scales have previously been validated against the original job strain questionnaires [22].

**Table 1:** Measurement of psychological demands, decision latitude and depressive symptoms

**Construct**	**Items**	**Response categories**	
		PUMA	IPAW
Psychological demands	Do you have to work very fast?	Always, often, some times, rarely, never/almost never	Often, Some times, Seldomely, Never/almost never
	Do you have enough time for your work tasks?	Always, often, some times, rarely, never/almost never	Often, Some times, Seldomely, Never/almost never
	Are contradictory demands placed on you at work?	Always, often, some times, seldomely, never/almost never	-
Decision latitude	Do you have the possibility of learning new things through your work?	To a very high extent, to a high extent, somewhat, to a low degree, to a very low degree	Often, Some times, Seldomely, Never/almost never
	Does your work require you to take the initiative?	To a very high extent, to a high extent, somewhat, to a low degree, to a very low degree	Often, Some times, Seldomely, Never/almost never
	Do you have any influence on what you do at work?	Always, often, some times, seldomely, never/almost never	Often, Some times, Seldomely, Never/almost never
	Is your work varied?	To a very high extent, to a high extent, somewhat, to a low degree, to a very low degree	-
	Do you have any influence on how you do your work?	-	Often, Some times, Seldomely, Never/almost never
	Does your work demand a high level of skill or expertise?	-	Often, Some times, Seldomely, Never/almost never
	Do you have to do the same thing over and over again?	-	Often, Some times, Seldomely, Never/almost never
Depressive symptoms (Mental health inventory)	During the past month, how much of the time were you a happy person?	All of the time, most of the time, a good bit of the time, some of the time, a little of the time, none of the time	Same as PUMA
	During the past month, how much of the time have you felt calm and peaceful?	All of the time, most of the time, a good bit of the time, some of the time, a little of the time, none of the time	Same as PUMA
	During the past month, how much of the time have you been a very nervous person?	All of the time, most of the time, a good bit of the time, some of the time, a little of the time, none of the time	Same as PUMA
	During the past month, how much of the time have you felt downhearted and blue?	All of the time, most of the time, a good bit of the time, some of the time, a little of the time, none of the time	Same as PUMA
	During the past month, how much of the time did you feel so down in the dumps that nothing could cheer you up?	All of the time, most of the time, a good bit of the time, some of the time, a little of the time, none of the time	Same as PUMA

Private life social support was operationalized as the number of life domains in which the respondent had one or more confidants and measured by asking the respondent: “is there someone you can really talk to about something personal which is important to you?” It was possible to indicate multiple domains and we defined private life social support from the sum of the following responses: yes, parents; yes, spouse/partner; yes, children; yes, family; yes, friends. We dichotomized private life social support into low (0–1 life domains with confidants) or high (2 or more domains with confidants). This dichotomization was conducted following considerations of the construct content and the distribution of respondents. We did not treat private life social support as a continuous variable to avoid assuming a linear relation between the number of domains with confidants and any buffering of high strain work. In the IPAW study, participants of wave 2 (our study baseline) were asked not to complete the section of the questionnaire including the measure of private life social support, if they had already participated in the study at wave 1. Consequently, for these participants we used the wave 1 score as our baseline measurement of private life social support.

Severe depressive symptoms were measured by the Mental Health Inventory (MHI-5) a five item scale from the Short-Form Health Survey [23]. The items of the MHI-5 are shown in Table 1. Although the scale was originally constructed to measure general mental health, a previous validation study found it an appropriate screening tool for mood disorders diagnosed according to the DSM-IV, including major depression and dysthymia [24]. We scored each item of the scale from 0–100 with higher scores indicating fewer symptoms, and calculated a mean score, if participants had data on three or more items. Following previous studies [25-27] we defined severe depressive symptoms as a mean score ≤52. This cut-off point has been found to have a 83% sensitivity and 65% specificity, compared to a clinical diagnosis of major depression [27].

Socioeconomic position at wave 2 was measured by national register data [28] on occupation, coded according to the International Standard Classification of Occupations version 88 [29] and linked to the questionnaire data using the unique personal identification number assigned all Danish residents [30]. Socioeconomic position was classified as low, intermediate or high following the European Socio-economic Classification [31,32]. Workplace social support was measured by two items asking the respondents how often they receive help and support from their manager and colleagues, respectively.

### Data analysis

Data were analyzed by logistic regression, including an indicator of study and organization as a strata-variable to account for clustering within the study- or organizational level. We used wave 2 as baseline for the analyses and excluded all participants with severe depressive symptoms at this wave. We then examined prospective associations between wave 2 high strain work and low private life social support, and onset of severe depressive symptoms measured at wave 3. Risk estimates were adjusted for potential confounding by sex, age, and occupational position as these are known for their association with depression and are also possibly related to high strain work [33,34]. Furthermore we adjusted for prior depressive symptoms (continuous score, wave 1) to account for bias due to negative affectivity [35]. As a sensitivity analysis we additionally adjusted for cohabitation (living with a partner or spouse, yes/no) which is also associated with depression [36]. This adjustment was not included in the main analysis to prevent collinearity between cohabitation and low private life social support. As a second sensitivity analysis, we adjusted for workplace social support in addition to the potential confounders included in the main analysis.

We present results as odds ratios (ORs) and adjusted absolute risks of onset, calculated by multiplying the reference group’s risk of onset by the adjusted odds ratio. Because sex might modify associations between work environment and mental health [37] we tested for interaction (departure from multiplicativity) between sex and the joint association of depressive symptoms with high strain work and low private life social support. As this interaction term was statistically non-significant (p = 0.66) we did not stratify analyses by sex.

In this article we examine effect-modification. Effect-modification may be defined as departure either from risk addivity or from risk multiplicativity [16]. The distinction between these types of effect-modification is not trivial, as similar stratum-specific relative risks (risk multiplicativity) may reflect different absolute risks (departure from risk additivity), when the risk rates of the outcome differ across strata of the effect-modifier [16]. Following the STROBE recommendations [38] we report risk estimates to assess both types of effect-modification, as outlined by Knol & VanderWeele [39]. We test for departure from additivity by including an interaction term in a linear probability model, and for departure from multiplicativity by including an interaction term in the logistic model. We base our conclusions regarding effect-modification on departure from additivity because such departure is particularly relevant from the clinical and public health perspectives and identifies groups who benefit most from exposure intervention [16,38-40]. All analyses were conducted in SAS version 9 (SAS Institute Inc., Cary, North Carolina).

### Study population

Table 2 shows the characteristics of the study participants. Most participants were women (75%), and of low socioeconomic position (56%). There were 168 participants with high strain work at baseline (16%) and 284 with low private life social support (26%). At follow up, 69 participants had onset of severe depressive symptoms (6%). The median follow up was 2.7 years (range 1.6-4.9) (data not shown in tables).

**Table 2:** Participant characteristics at baseline

	**N (%)**	**Mean (SD)**
**Total**	1074 (100)	
*Demographics*		
**Women**	804 (74.9)	
**Age**		45 (8.8)
**Socioeconomic position**		
High	253 (23.6)	
Intermediate	224 (20.9)	
Low	597 (55.6)	
*Exposure and outcome*		
**High strain work**	168 (15.6)	
**Incident severe depressive symptoms**	69 (6.4)	
**Low private life social support**	284 (26.4)	
**Private life social support, domains with confidants**		
0	30 (2.8)	
1	254 (23.7)	
2	320 (29.8)	
3	282 (26.3)	
4	145 (13.5)	
5+	43 (4.0)	
*Joint exposure high strain work and low private life social support*		
**High strain work**		
low private life social support	47 (4.4)	
high private life social support	129 (12.0)	
**Not high strain work**		
low private life social support	237 (22.1)	
high private life social support	661 (61.6)	

## Results

Table 3 shows the main associations between high strain work and low social support and the development of severe depressive symptoms. Neither high strain work nor low private life social support statistically significantly predicted onset of depressive symptoms, although both odds ratio’s (OR) were above one.

**Table 3:** Depressive symptoms in relation to high strain work and low private life social support (main effects)

	**OR**^**a **^**(95% CI)**	**P-value**
High strain work, yes vs. no	1.72 (0.97-3.07)	0.06
Private life social support, low vs. high	1.32 (0.74-2.35)	0.35

^a^Adjusted for sex, age, occupational position, and prior depressive symptoms.

**Figure 1:**
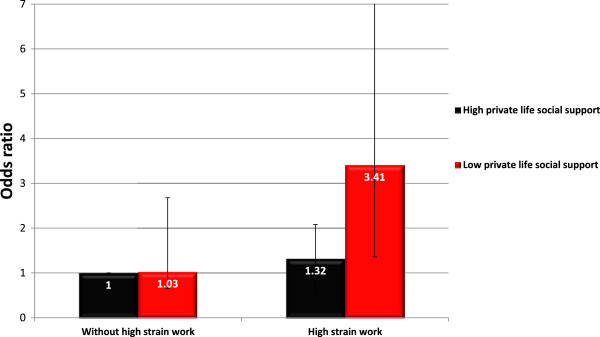
Odds ratio for depressive symptoms with exposure to high strain work and private life social support.

Table 4 shows the risk of onset of severe depressive symptoms in relation to the joint exposure to high strain work and low private life social support. The results are visualized in Figure 1. Compared to individuals without high strain work and with high social support, the risk of onset of severe depressive symptoms was over three times greater for individuals with high strain work and low social support (OR = 3.41, 95% CI: 1.36-8.58). In absolute terms, the risk of onset of depressive symptoms was 17.4% in individuals with high strain work and low private life social support, whereas it was 5.1% in the reference group. The separate exposure to either high strain work or low private life social support was not associated with a statistically significantly increased risk. The stratified analysis yielded similar results; the risk estimate for high strain work was larger in the presence of low private life social support (OR = 3.50, 95% CI: 1.25-9.80) than in the presence of high private life social support (OR = 1.24, 95% CI: 0.61-2.53). When testing for departure from additivity between the effects of high strain work and low social support on severe depressive symptoms, however, the p-value was not statistical significance (p = 0.18). The p-value for departure from multiplicativity was 0.12. Neither adjustment for cohabitation nor adjustment for workplace social support changed this result substantially (data not shown).

**Table 4:** Depressive symptoms related to joint exposure to high strain work and private life social support (interactive effects)

	**Not high strain work**			**High strain work**			**High strain work (yes vs. no), within strata**	**P-value, within strata**	**P-value, overall**
	N/N cases	OR^a^ (95% CI)	Incidence rate^a^, cases per 100	N/N cases	OR (95% CI)	Incidence rate^a^, cases per 100	OR (95% CI)		
Private life social support, high	661/34	1 (reference)	5.1	129/13	1.32 (0.65-2.68)	6.7	1.24 (0.61-2.53)	0.55	
Private life social support, low	237/13	1.03 (0.51-2.08)	5.3	47/9	3.41 (1.36-8.58)	17.4	3.50 (1.25-9.80)	0.02	
									0.07

^a^Adjusted for sex, age, occupational position, and prior depressive symptoms.

## Discussion

Employees with high strain work and low private life social support were over three times more likely to develop severe depressive symptoms during the 2.7 years of follow up of this study compared to employees with no high strain work and high private life social support. There was no statistically significantly increased risk for employees with high strain work and high private life social support or for employees with low private life social support and no high strain work.

Our findings suggest a role of private life social support as a buffer of high strain work, although we acknowledge that the interaction term did not reach statistical significance. Even though evidence is building concerning the role of workplace social support as a buffer of the effects of high strain work on mental health [15], research about the role of private life social support is scarce. A previous study from Stansfeld et al. [41] found no evidence for a modifying role of private life social support. That study examined whether the associations between elements of the job strain model (job demands and decision latitude) and poor mental health were modified by private life social support. They found no statistically significant interactions, defined as departure from multiplicativity, but did not report risk estimates for the assessment of interaction, due to the statistical non-significance. Hence, it was impossible to assess the extent to which the patterns of risk estimates supported effect-modification in their article. Given the extensive demands on statistical power for the detection of effect-modification [42], analyses in larger samples are needed to shed light on the potential role of private life social support as a buffer of the association between high strain work and mental health.

We operationalized private life social support as the availability of confidants within different life domains, measured by a single item. Although the availability of others to confide in is a central aspect of social support [43], social support is a complex and multidimensional construct encompassing both structural and functional elements [44]. The structural dimension, for which we had a single indicator in the present study, denotes the social network, which provides the opportunity for social support. The network may be characterized by its size, density, and range [45] and the individual social ties of the network may further be characterized by their reciprocity and the frequency of contact [45]. The functional aspect - the social support received through the ties - may be instrumental, informational, appraisal or emotional [45]. Although we did not measure the functional aspect of social support in the present study, confidants are regarded an important source of emotional support [45]. Consequently, emotional support may be particularly important for the interpretation of our findings. Emotional support may consist of signaling empathy and understanding, listening, and showing sympathy, thus strengthening the individual’s sense of self-esteem, mattering and belonging [13]. Also, emotionally supportive behaviors help validate the individual’s feelings and concerns, and enable emotion focused coping such as ventilation [13].

Our definition of low private life social support, reporting zero or one life domains with confidants, was based partly on the distribution of the respondents. Although it might be considered more clear cut to define low private life social support as having zero confidants, this was unfortunately not possible given a low number of individuals in this group (n = 30). We argue, however, that our definition indicates a narrow range of close ties in the social network. Such narrow range likely puts the individual at risk of lacking social support when needed; in the case of zero domains because no confidants are available to provide the support, and in the case of one domain because drawing on social support may lead to its depletion, especially in conditions of chronic strain [43]. A narrow social network provides fewer alternative sources of social support, possibly leaving the individual without the needed social support. Conversely, depression may also undermine social relations [46], however such reverse causality was addressed in the present analyses by applying a prospective study-design. As high strain work likely is a chronic rather than acute stressor, the availability of different sources of social support may be particularly important.

Some additional methodological issues, apart from the single item measurement of low private life social support, must be considered in interpreting the findings of this study. We measured both exposures and outcome by self-report. This methodology has been questioned, as associations may be biased upwards due to common method variance [7,47,48]. To address this bias, we adjusted our results for previous depressive symptoms, which also served as an indicator of negative affectivity [21]. Although such analyses may be considered overly conservative, if the previous mental health state of the employee was a consequence of work environment factors, we consider this adjustment a substantial strength of our analyses, as the remaining associations indicate that our findings are not explained by common method variance bias.

Another important concern is the generalizability of our findings. Although our study population was cross-occupational it was not representative of the Danish working population. More than half of the participants (55.6%) were classified as having low socioeconomic position, reflecting the organizations from which they were recruited [17,18] and, most participants were women. It is likely that these population characteristics explain the relatively high prevalence of severe depressive symptoms in the sample. The baseline prevalence was 8.8%; in comparison, the prevalence of major depression in the Danish general population has been estimated as 2-5% [25,49,50]. Consequently, it is possible that the generalizability of our findings is limited by the characteristics of the sample.

The measurement of high strain work did not apply the full measurement of the demands-control model, as operationalized by the Job Content Questionnaire [51]. However, we believe that this is unlikely to be a major concern, because a validation study comparing the included items with the items of the full instrument showed satisfactory performance of our items [22].

The average follow up of this study was over 2 years. During this period both the working conditions of the individual and their level of private life social support may have changed, causing misclassification of these factors. In the present study we could not account for such changes. If such changes were non-differential in relation to the joint exposure to high strain work and low private life social support, they will have led to an underestimation of the effects. If the changes were differential they may have been mediating factors rather than confounders.

The follow up outcome measurement was conducted at a fixed time. Consequently, some participants may have developed depressive symptoms during the follow up period but not been considered cases in the present study, if they were in remission at the time of measurement at the end of the follow up. Hence, the reported risks of onset may be underestimated.

We excluded all cases with severe depressive symptoms at baseline (wave 2) and adjusted for depressive symptoms in the data collection prior baseline (wave 1). However, we did not have information on depressive symptoms in life phases preceding wave 1 or between the study waves.

Finally, the sample size of the study was relatively small for studying interactions, resulting in wide confidence limits for some estimates. This issue also precluded us from exploring the multi-level effects which could conceivably exist, as the data were collected from workplaces nested within organisations. As effect-modification studies require substantial statistical power [42], and our finding showed a suggestive albeit not statistically significant effect-modification we propose that studies of larger populations are needed on the interplay between work stressors, private life social support, and depressive symptoms.

## Conclusions

The findings of this study suggest that high strain work may be particularly detrimental to the mental health of employees with weaker private life social support. Among these employees, the adjusted risk of severe depressive symptoms at follow up was 17%. The implications of these findings are two-fold: first, employees with high strain work and weak private life social support may be a high risk group for developing depressive symptoms, and ensuring adequate treatment for this group is an important concern. Second, the prevention or elimination of high strain work may be particularly important in individuals with weaker private life social support, or conversely, the strengthening of social ties may be particularly important in individuals exposed to high strain work. These findings may guide public health initiatives aimed at preventing depression in the employed population. The test for effect-modification, was, however not statistically significant. In light of this statistical non-significance, and given the extensive demands on statistical power for the detection of effect-modification [42], larger sample sizes are needed to shed further light on the potential role of private life social support as a buffer of the association between high strain work and mental health.

## Competing interests

The authors declare that they have no competing interests.

## Authors’ contributions

IM contributed to the design and conception of the study, conducted the data analysis, and drafted the manuscript. AJ contributed to the data interpretation and critically revised the manuscript. MB and MN were responsible for the data collection and critically revised the manuscript. RR contributed to the study design and conception, the interpretation of data, and critically revised the manuscript. All authors have read and approved the final manuscript.

## Pre-publication history

The pre-publication history for this paper can be accessed here:

http://www.biomedcentral.com/1471-2458/14/698/prepub
